# A Prospective Hospital Study to Evaluate the Diagnostic Accuracy of Rapid Diagnostic Tests for the Early Detection of Leptospirosis in Laos

**DOI:** 10.4269/ajtmh.17-0702

**Published:** 2018-02-26

**Authors:** Sabine Dittrich, Latsaniphone Boutthasavong, Dala Keokhamhoung, Weerawat Phuklia, Scott B. Craig, Suhella M. Tulsiani, Mary-Anne Burns, Steven L. Weier, David A. B. Dance, Viengmon Davong, Manivanh Vongsouvath, Mayfong Mayxay, Rattanaphone Phetsouvanh, Paul N. Newton, Kate Woods

**Affiliations:** 1 Microbiology Laboratory, Lao-Oxford-Mahosot Hospital-Wellcome Trust Research Unit, Mahosot Hospital, Vientiane, Lao PDR;; 2 Nuffield Department of Medicine, Centre for Tropical Medicine and Global Health, University of Oxford, Oxford, United Kingdom;; 3 Faculty of Science Health, Education and Engineering, University of the Sunshine Coast, Sippy Downs, Australia;; 4 Queensland Health Forensic and Scientific Service, WHO Collaborating Centre for Reference and Research on Leptospirosis, Brisbane, Australia;; 5 Section for Global Health, Department of Public Health, Copenhagen Centre for Disaster Research, University of Copenhagen, Copenhagen, Denmark;; 6 Faculty of Health, Queensland University of Technology, Brisbane, Australia;; 7 Faculty of Tropical and Infectious Diseases, London School of Hygiene and Tropical Medicine, London, United Kingdom;; 8 Faculty of Postgraduate Studies, University of Health Sciences, Vientiane, Lao PDR;; 9 National Infection Service (NIS), Public Health England (PHE), London, United Kingdom

## Abstract

Leptospirosis is a globally important cause of acute febrile illness, and a common cause of non-malarial fever in Asia, Africa, and Latin America. Simple rapid diagnostic tests (RDTs) are needed to enable health-care workers, particularly in low resource settings, to diagnose leptospirosis early and give timely targeted treatment. This study compared four commercially available RDTs to detect human IgM against *Leptospira* spp. in a head-to-head prospective evaluation in Mahosot Hospital, Lao PDR. Patients with an acute febrile illness consistent with leptospirosis (*N* = 695) were included in the study during the 2014 rainy season. Samples were tested with four RDTs: (“Test-it” [Life Assay, Cape Town, South Africa; *N* = 418]; “Leptorapide” [Linnodee, Ballyclare, Northern Ireland; *N* = 492]; “Dual Path Platform” [DPP] [Chembio, Medford, NY; *N* = 530]; and “SD-IgM” [Standard Diagnostics, Yongin, South Korea; *N* = 481]). Diagnostic performance characteristics were calculated and compared with a composite reference standard combining polymerase chain reaction (PCR) (*rrs*), microscopic agglutination tests (MATs), and culture. Of all patients investigated, 39/695 (5.6%) were positive by culture, PCR, or MAT. The sensitivity and specificity of the RDTs ranged greatly from 17.9% to 63.6% and 62.1% to 96.8%, respectively. None of the investigated RDTs reached a sensitivity or specificity of > 90% for detecting *Leptospira* infections on admission. In conclusion, our investigation highlights the challenges associated with *Leptospira* diagnostics, particularly in populations with multiple exposures. These findings emphasize the need for extensive prospective evaluations in multiple endemic settings to establish the value of rapid tools for diagnosing fevers to allow targeting of antibiotics.

## INTRODUCTION

Leptospirosis is an important zoonotic disease worldwide, with its frequency and severity increasingly recognized.^1,2^ It has also been shown to be a significant cause of meningoencephalitis in Laos and Thailand.^3^ Leptospirosis is caused by *Leptospira* spp. spirochetes contracted by humans through exposure to environments contaminated by urine of infected mammals.^2^ It is estimated that ∼853,000 people are infected and 48,000 die annually.^4^ Most of the cases occur in the tropics, particularly in urban slums and rural areas where people are exposed to contaminated water.^2^ The clinical presentation of leptospirosis is often nonspecific, and as the organism does not grow well in conventional blood cultures, diagnosis is difficult, requiring sophisticated serological and molecular tests. However, vast areas of the tropics where leptospirosis is endemic have extremely limited diagnostic laboratory capacity.^5^ Even where the laboratory capacity exists, diagnosis using specific culture or serological microscopic agglutination test (MAT) methods^2^ requires considerable expertise that is not widely available, and results are only available weeks after the initial clinical presentation. At this point, no clear guidance by international bodies such as the World Health Organization (WHO) exists as to which test is recommended for acute detection. Conventionally, the observation of a 4-fold rise between the acute and convalescent sample is considered a clear indication of an acute infection and is therefore considered the gold standard; however, a recent modeling analysis has highlighted the pitfalls of this approach.^6^ Several manufacturers have developed rapid diagnostic tests (RDTs) for use at the bedside or point-of-care^7^ of which so far, none has been approved by a stringent regulatory authority. The simplicity and relatively low cost of these tests make them potentially well suited for use in resource-poor settings with limited laboratory and human capacity, as has been achieved with malaria RDTs. Evaluations of RDTs detecting IgM against *Leptospira* spp. antigens have been conducted, and their diagnostic characteristics have been reported to vary between areas of low and high endemicity.^8^ Goris et al.^8^ reported 69% sensitivity and 96% specificity for the LeptoTek lateral flow test when used on admission sera in a Dutch population, whereas the same test used in a Southeast Asian hospital setting (Lao PDR) had only 45% sensitivity and 75% specificity.^9^ These differences are very important, as a test may be well suited to one setting but not to another. It is likely that the differences, particularly for specificity, are mainly due to background antibody levels in patients who have had multiple exposures to the pathogen, similar to the challenges faced with *Orientia tsutsugamushi* (scrub typhus) diagnosis in endemic areas.^10^

To understand these challenges and identify an RDT that is suitable for use in an endemic setting for populations repeatedly exposed to the pathogen, on-site evaluations are necessary. Our study aimed to compare the diagnostic characteristics of four RDTs for leptospirosis to guide local and regional health authorities in their search for a suitable diagnostic tool to incorporate into rapid diagnostic panels in the region.

## MATERIALS AND METHODS

### Study population.

Consecutive patients were enrolled in the 2014 rainy season in Mahosot Hospital (longitude 17°96′04·4″N, latitude 102°61′19·1″E) in Vientiane, Lao PDR (Laos), as part of an ongoing febrile illness study.^11^ Patients admitted to any ward with fever < 1 month (either history of fever during this illness or documented fever > 38.0°C by axillary temperature) plus at least one of the following symptoms (indicative of leptospirosis or typhus): headache, rash, eschar, myalgia, arthralgia, lymphadenopathy, meningitis, encephalitis, respiratory symptoms (cough, crepitations, respiratory rate > 20/minutes), clinical jaundice, or acute renal failure (creatinine > 120 μmol/L) were eligible.

### Ethics statement.

Study patients provided written informed consent. In case of children, a parent or guardian provided informed consent on their behalf. Ethical approval for all investigations was granted by the Oxford Tropical Research Ethics Committee, University of Oxford, United Kingdom, and the National Ethics Committee for Health Research, Laos. All samples were anonymized using a unique identifier in all procedures and analysis.

### Testing procedure.

All RDTs were performed on fresh serum within 24 hours of receipt in the Mahosot Hospital Microbiology Laboratory. Sera were refrigerated before analysis. The same three laboratory technicians performed all tests for all patients, blinded to each other’s results. Four RDTs, all detecting *Leptospira* IgM, were compared using only the admission sample: “Test-it” (Life Assay, Product Code: LEPTO01, South Africa; *N* = 418), “Leptorapide” (Linnodee, owslips.com/linnodee/ordering.html, Northern Ireland; *N* = 492), “Dual Path Platform” (DPP) (Chembio, Medford, NY, not commercially available; *N* = 530), and “SD-IgM” (Standard Diagnostics/Alere, not available at the time of writing, South Korea; *N* = 481). Serum (5 or 10 μL, as appropriate) was used for all assays and the tests were performed according to the manufacturers’ instructions. Different numbers are because of varying numbers of donated tests and other logistical considerations (e.g., start of test inclusion due to test availability at Mahosot Hospital).

### Reference diagnostics.

Leptospiral MATs were performed and interpreted by the WHO Collaborating Center for Reference and Research on Leptospirosis, Australia. A 4-fold increase between admission and convalescent samples was considered “evidence of acute infection,” whereas a 2-fold increase/decrease or an admission titer ≥ 1:400 was considered “evidence of recent infection.” Only a subset of patients had both admission and follow-up sample available for MAT testing (*N* = 248) and therefore samples positive only at admission as well as a 4-fold rise were combined for accuracy analysis. Quantitative real-time PCR (qPCR) was performed on 687/695 (98.8%) patients using DNA extracted from serum (sample taken at presentation) to detect the *rrs* gene according to previously described protocols.^12^ Cultures were performed on blood clots, remaining after removal of serum, as described previously.^3^

### Analysis.

Data were analyzed using STATA 10.0 (Stata Corp., College Station, TX) and MedCalc for Windows, version 15.0 (MedCalc Software, Ostend, Belgium). Direct (qPCR, culture) and indirect (MAT, acute, and recent infection) diagnostic tests were combined to give a composite^13^ reference standard (unless stated otherwise) which was used to calculate the diagnostic accuracy values (sensitivity, specificity, and positive and negative predictive values [PPV and NPV]). To assess the interobserver agreement, kappa was calculated between the three readers for the subset of tests that had been read by all readers (1–3). Diagnostic performance characteristics (sensitivity/specificity/NPV/PPV) for the different tests were based on results obtained from only Reader 1 as all tests had been read by this reader, whereas only a subset was read by Reader 2 and 3. STARD checklist attached in supplement ST1.

## RESULTS

### Patient characteristics.

**Figure 1.**
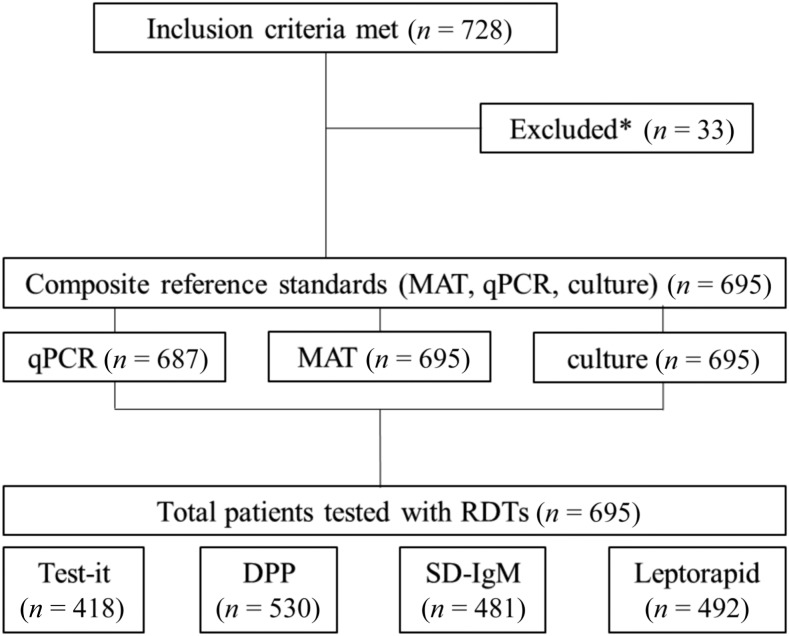
Flow of participants, reference, and investigated tests. * Excluded because of lack of sample for reference testing.

Between May 2014 and January 2015, 728 consecutive patients met the inclusion criteria (728/1,324, 55.0%). Of these, 33 were excluded from analysis, as insufficient sample for any of the reference testing (MAT and/or PCR) was available. Hence, the final number of patients included in the analysis was 695 (Figure 1). The majority were men (407/695, 58.6%) with a median age of 39 years (range: 0.5–92). Patients presented with a median of 5 days of fever (interquartile range: 3–7). Of all patients included, 39/695 (5.6%) were positive using the composite reference standard (MAT/qPCR/culture); 12/695 (1.7%) were positive for leptospirosis by qPCR alone and 27/695 (3.9%) by MAT (“evidence of acute infection”: 10/248; “evidence of recent infection”: 37/695). No patient was only positive by culture (n[culture] = 4). The overall positivity rate of the different RDTs ranged from 5.80% to 38.1% (“Test-it”: 154/418, 36.8%; “SD-IgM”: 28/481, 5.8%; “DPP”: 202/530, 38.1%; and “Leptorapide”: 117/492, 23.8%) in the tested subset.

### Diagnostic performance characteristics.

**Figure 2.**
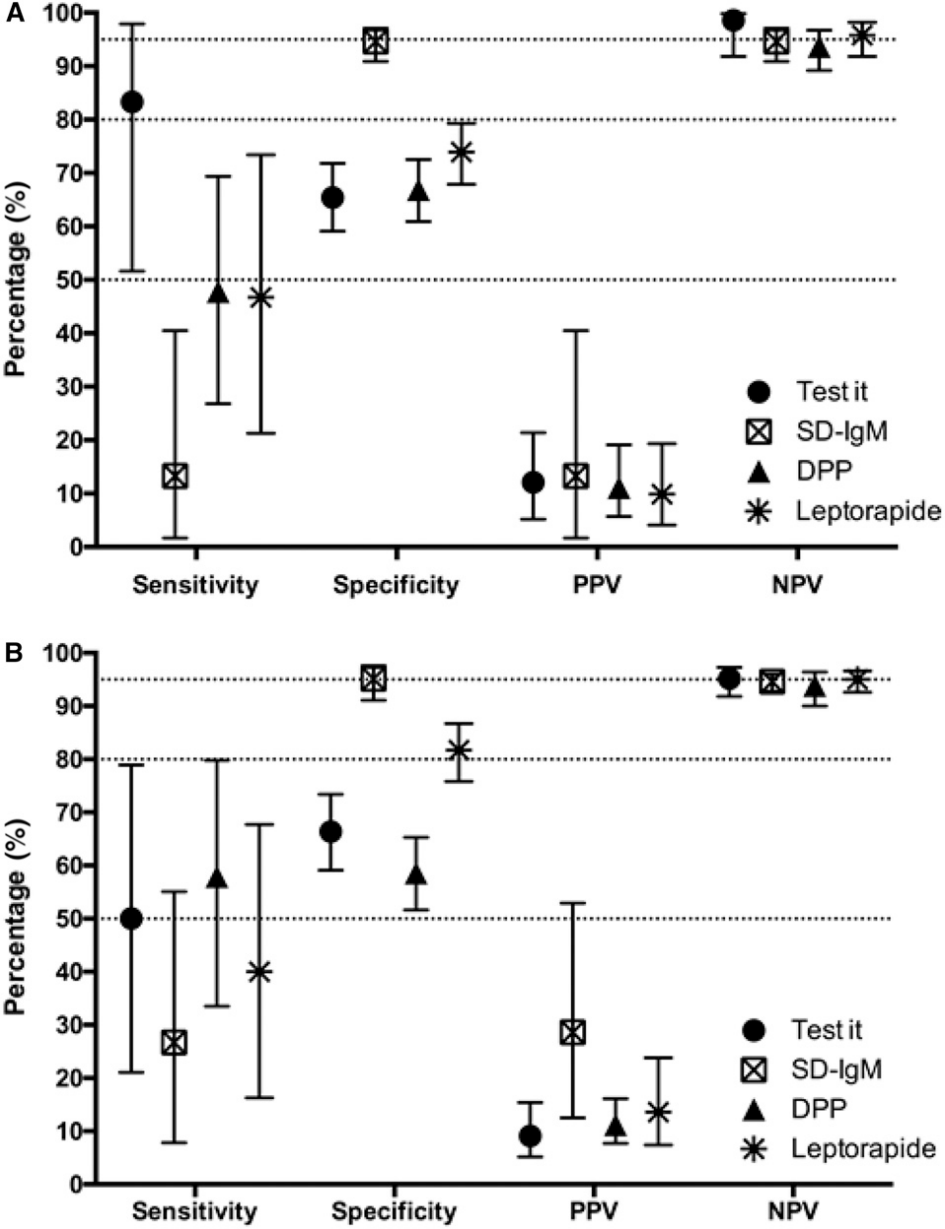
Diagnostic characteristics for patients with ≤ 5 or > 5 days of fever before presentation. Rapid diagnostic test (RDT) results are compared with a composite reference standard (MAT/culture/qPCR) according to fever duration. (**A**) Sensitivity (95% CI), specificity (95% CI), PPV (95% CI), and NPV (95% CI) are shown for all RDTs with a subset of patients who presented with five or less days of fever (Test-it: *N* = 223; SD-IgM: *N* = 255; DPP: *N* = 289; and Leptorapide: *N* = 260). (**B**) Sensitivity (95% CI), specificity (95% CI), PPV (95% CI), and NPV (95% CI) are shown for all RDTs with a subset of patients who presented with more than 5 days of fever (Test-it: *N* = 191; SD-IgM: *N* = 217; DPP: *N* = 229; and Leptorapide: *N* = 226). All presented results are based on Reader 1. Dotted lines are included to highlight 50%, 80%, and 95%. CI = confidence intervals; DPP = dual path platform; MAT = microscopic agglutination test; NPV = negative predictive value; PPV = positive predictive value.

The diagnostic accuracy and respective confidence intervals (CIs) of the RDTs ranged considerably between the different tests when using the composite gold standard (Table 1, Figure 2). For Reader 1, sensitivity ranged from 17.9% to 63.6% between RDTs, with the “Test-it” assay displaying the highest diagnostic sensitivity. Sensitivities ranged from 37.5% to 66.7% when comparing RDT results with the small subset of patient with “evidence of acute infections” (Test-it: 4/6, 66.7%; SD: 3/8, 37.5%; DPP: 6/9, 66.7%; and Leptorapide: 5/8, 62.5%). When using the composite gold standard, the range of specificities was also wide (62–97%) for all investigated RDTs, with the SD-IgM assay displaying the highest diagnostic specificity (Figure 2).

**Table 1:** Diagnostic characteristics by test and reader using a composite reference standard (MAT/culture/qPCR) including the agreement between readers estimated using kappa

Assay	Parameter	Reader 1* (95% CI)	Reader 2† (95% CI)	Reader 3‡ (95% CI)	Kappa
Test-it	Sensitivity	71.0% (41.9–91.6)	62.5% (24.5–91.5)	80.0% (44.4–97.5)	0.56
	Specificity	64.6% (59.8–69.3)	69.5% (63.2–75.4)	48.5% (41.3–55.7)	
SD IgM	Sensitivity	21.1% (6.1–45.6)	12.5% (0.3–52.7)	26.7% (7.8–45.4)	0.73
	Specificity	94.8% (92.6–96.7)	95.7% (92.3–97.9)	93.9% (90.4–96.8)	
DPP	Sensitivity	35.0% (15.4–59.2)	60.0% (14.7–94.7)	42.1% (20.3–66.5)	0.81
	Specificity	62.1% (57.7–66.4)	54.5% (44.2–64.4)	58.7% (54.5–63.9)	
Leptorapide	Sensitivity	47.4% (24.5–71.1)	25.0% (3.2–65.1)	53.3% (26.6–78.7)	0.96
	Specificity	77.2% (73.1–80.9)	85.8% (80.7–90.1)	66.9% (60.8–72.7)	

CI = confidence intervals; DPP = dual path platform; MAT = microscopic agglutination test. Reader 1 read all tests for all patients included in the study. Reader 2 and 3 read a subset of the tests in varying order after Reader 1. Kappa was calculated on the subset that was read by all readers (“Test-it”: 90, “SD IgM”: 63, “DPP”: 78, and “Leptorapide”: 63).

* Reader 1: “Test-it”: 418, “SD-IgM”: 480, “DPP”: 530, and “Leptorapide”: 492.

† Reader 2: “Test-it”: 242, “SD-IgM”: 332, “DPP”: 106, and “Leptorapide”: 242.

‡ Reader 3: “Test-it”: 206, “SD-IgM”: 411, “DPP”: 474, and “Leptorapide”: 272.

None of the tests performed with a sensitivity and specificity of > 80% for detecting leptospirosis in admission samples, when comparing the tests with the composite reference standard. The “Test-it” RDT performed better in patients reporting 5 days of illness or less, whereas sensitivity dropped in patients presenting later (Figure 2). The DPP and SD assays performed better for patients reporting more than 5 days of illness, but all tests had large 95% CIs because of small number of positives (Figure 2). The performance of the Leptorapide test was similar at any day of presentation with sensitivities never reaching 50%. None of the tests showed significantly better sensitivity performance, with all CIs overlapping. In contrast, the SD-IgM test showed significantly better specificity in all patients, regardless of days of illness. PPVs for all investigated tests were very low with many false positives regardless of the manufacturer, test comparator, or reported days of illness (Figure 2).

### Interobserver variability.

For the subset of tests that were read by all readers, the diagnostic accuracy values varied greatly, indicating that readers interpreted results differently (Table 1). Sensitivity ranged by 10–30% depending on the assay, when different laboratory technicians read a subset of the results. The least concordance between readers was recorded for the lateral flow–based “Test-it” assay (kappa: 0.56), whereas the agglutination-based “Leptorapide” assay (kappa: 0.96) was most consistently interpreted by the three readers. Of the three lateral flow–based tests, the DPP had the highest agreement (kappa: 0.81).

## DISCUSSION

Given the global environmental presence of *Leptospira* spp. and that they have been identified as an important cause of fever in many large non-malarial fever studies,^14–16^ a simple, rapid diagnostic tool for diagnosing leptospirosis could have a large impact on patient care globally. In this study, we evaluated four RDTs which all detect anti-*Leptospira* IgM. The “Test-it” and “SD-IgM” are designed as simple lateral flow tests, whereas the “Leptorapide” is an agglutination test and the “DPP” is a lateral flow test with a unique dual path (DPP) technology.^8,17^ Although the three cassette-based tests represent familiar, supposedly simple-to-interpret, platforms, there was considerable interobserver variability between the three readers in this study. This was less the case for the Leptorapide test, which is an agglutination test. It is conceivable that in some cases, a delay in reading results may have occurred between the three readers that could have contributed to the observed inter-reader variability due to fading/intensifying of bands over time. Although this observation might not be representative because of the very small sample size, it is important to follow our findings up with more research to support product improvement efforts. When using a composite reference standard as comparators for the diagnostic accuracy assessment, no clearly superior RDT could be identified. The DPP assay performed consistently regardless of the days of illness with a sensitivity between 50% and 60% and specificity around 70%, which is in line with what was previously published for mild leptospirosis cases at admission as well as healthy slum habitants.^18^ In comparison to previously published sensitivity and specificity of more than 90%,^17^ we found that the “Leptorapide” assay showed a lower sensitivity (< 50%) and specificity (∼80%) combined with an NPV of ∼95%. The “Test-it” assay had a high sensitivity of ∼80% in patients with less than 5 days of fever and the specificity of the test was low at ∼70%. Earlier evaluations^3,6^ of this assay reported a higher specificity, and the difference can likely be explained by the fact that our study population consisted of individuals who had multiple episodes of exposure to *Leptospira* spp. It must be noted that one additional reason for the different results in different studies for all the tests could also be due to batch variations related to substandard manufacturing.

One significant limitation of our study is that not all tests were performed on all samples because of logistical challenges. This might have influenced the comparability of results between tests. In addition, no extensive comparison was drawn to severity of infections as the study aim was to understand the diagnostic usability to identify leptospirosis in the general population before progressing to severe disease.

The very low sensitivity of the SD-IgM assay makes it unsuitable for use as single diagnostic test in Laos, unless combined with a secondary test. It could be envisioned that combining a high sensitive, but low specificity test with a low sensitivity but high specificity test to provide more accurate diagnosis to patients. A similar “screen-and-confirm” approach is taken with human immunodeficiency virus or Hepatitis C where positive high-sensitive screening tests are followed up with more specific confirmatory tests.^19,20^ One could hypothesize that screening with the “Test it” or DPP test and retesting all positives with the SD-IgM assay could provide more confidence in the diagnosis where molecular testing or MAT is not possible.

The data form this study confirm that local validations are important to understand the performance of a test in a population with particular health-seeking behavior or disease epidemiology.^21^ Furthermore, to allow wider decentralization of tests in the future, the expansion of sample types to whole blood would very much improve the usability of the test beyond central facilities. In conclusion, none of the tests evaluated in this study showed both sensitivity and specificity > 90%, which is disappointing but not surprising, given previous reports from endemic areas, including Laos.^9^ This is particularly important for diagnostic assays that detect the host–antibody response rather than directly detecting the pathogen, underlining the need for combined antigen/antibody detection or other improvements in the testing algorithm such as screen and confirm, where possible.
